# Unveiling Ultra-High Ionic Conductivity in W‑Doped Na_3_SbS_4_: Grain Boundary Effects and Pure Bulk Transport

**DOI:** 10.1021/jacs.5c05842

**Published:** 2025-05-29

**Authors:** Jana Königsreiter, Bernhard Gadermaier, H. Martin R. Wilkening

**Affiliations:** Institute of Chemistry and Technology of Materials (NAWI Graz), 27253 Graz University of Technology, Stremayrgasse 9, Graz 8010, Austria

## Abstract

W-doped Na_3_SbS_4_ is a promising solid electrolyte for all-solid-state sodium batteries, exhibiting a sodium (Na^+^) ionic conductivity higher than 30 mS cm^–1^ (A. Hayashi, N. Masuzawa, S. Yubuchi, F. Tsuji, C. Hotehama, A. Sakuda, M. Tatsumisago, *Nat. Commun.* 2019, 10, 5266). This exceptional conductivity arises primarily from the introduction of sodium ion vacancies (V′_Na_) via supervalent substitution of Sb^5+^ with W^6+^. Using low-temperature impedance spectroscopy down to *T* = 113 K (−160 °C), we demonstrate that previously reported room temperature conductivities of Na_2.9_Sb_0.9_W_0.1_S_4_ are influenced by grain boundary resistances, which can only be effectively separated from the total conductivity at such low temperatures. Our results indicate that the pure Na^+^ bulk conductivity can reach 96 mS cm^–1^ (D_σ_ = 0.98 × 10^–10^ m^2^ s^–1^) at room temperature, as extrapolated from accurately measured low-temperature data (1.8 mS cm^–1^ at –130 °C). Our study suggests that further minimizing detrimental grain boundary effects enables extraordinarily fast long-range Na^+^ ion transport in this sulfide.

## Graphical abstract

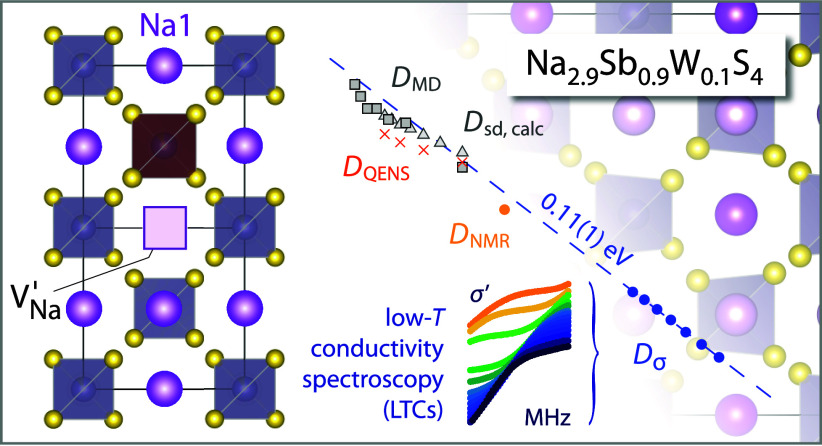

## Introduction

The transition to renewable energy sources is essential to mitigate climate change and reduce anthropogenic CO_2_ emissions. A key challenge in this transition is the development of efficient energy storage technologies that can effectively accommodate the intermittent nature of solar and wind power. All-solid-state batteries with lithium-ion (Li^+^) or sodium ion (Na^+^) charge carriers are among the most promising candidates for next-generation energy storage.^1,2^

A critical component in such batteries is the solid electrolyte,^3–8^ which must exhibit exceptionally high ionic conductivity to enable efficient ion transport while maintaining both electrochemical and mechanical stability.^9–13^ In this context, sodium-based solid electrolytes,^7–9^ such as Na_3_SbS_4_-type materials,^14–21^ have recently attracted attention due to their remarkable Na^+^ conductivity and the greater natural abundance of sodium compared to lithium, making Na-based batteries a more sustainable and cost-effective alternative. Several sodium-ion conductors have already been extensively studied,^8,15^ including Na-superionic conductors (NASICON)^8,22^ and Na-β″-alumina,^2,23,24^ both of which exhibit high room-temperature Na^+^ conductivity and have been considered for solid-state sodium battery applications.^2,8^ While these materials have shown very promising transport properties, W-doped Na_3_SbS_4_ stands out^16,17^ due to its exceptionally high ionic conductivity, reaching values higher than 30 mS cm^–1^ at room temperature, that is, comparable to or even exceeding the best Li^+^ ion-conducting thiophosphate electrolytes.^25–28^

So far, no studies have directly reported the pure Na^+^ bulk conductivity in W-doped Na_3_SbS_4_. Instead, we believe that previously published values,^16^ as high as (41 ± 8) mS cm^–1^ for Na_2.9_Sb_0.9_W_0.1_S_4_,^17^ represent total conductivities that include potential ion-blocking effects from grain boundary regions. Conductivities on the order of 10 mS cm^–1^ or higher correspond to electrical relaxation frequencies in the GHz range. Consequently, capturing the full electrical response — whether through conductivity isotherms or Nyquist plots, which depict the imaginary versus real part of the complex impedance — requires measurement equipment that is unaffected by any artifacts from stray capacitances^29^ and capable of precisely recording impedances at GHz frequencies and beyond. For instance, our group has successfully demonstrated such high-frequency impedance measurements for NASICON-type materials,^22,30^ F^–^ anion conductors^31^ and, quite recently, for Na-β″-alumina.^23^

An alternative approach to overcoming this challenge is to reduce the electrical relaxation frequency (ω_e_) by slowing down ionic mobility through cooling.^32–34^ Lowering the temperature shifts ω_e_ into the MHz range or even kHz range, where broadband impedance spectroscopy can more accurately probe the intrinsic transport properties. Following this approach, we investigated whether low-temperature broadband impedance spectroscopy could provide direct insight into the bulk conductivity of W-doped Na_3_SbS_4_. Our findings confirm that previously reported conductivities were indeed influenced by grain boundary resistances and that the intrinsic Na^+^ bulk conductivity, when extrapolated to room temperature, is considerably higher than assumed. These results suggest that further optimization of grain boundary properties could enable even faster long-range ion transport in Na_3_SbS_4_-type electrolytes, further advancing the development of efficient all-solid-state sodium batteries.

The crystal structure of Na_3_SbS_4_ is shown in Figure 1. Replacing Sb^5+^ with W^6+^ introduces Na^+^ vacancies V′_Na_ facilitating rapid sodium exchange processes that enable efficient, macroscopic ionic transport along the SbS_4_-network.^16,35–37^ Although the average structure is reported to be cubic at sufficiently high W-contents and depending on the synthesis method,^17,19,38^ the local symmetry remains tetragonal even in Na_2.9_Sb_0.9_W_0.1_S_4_ as has been convincingly demonstrated by Zeier and coworkers via, e.g., X-ray diffraction, pair distribution function analysis and static ^121^Sb NMR.^36^

**Figure 1:**
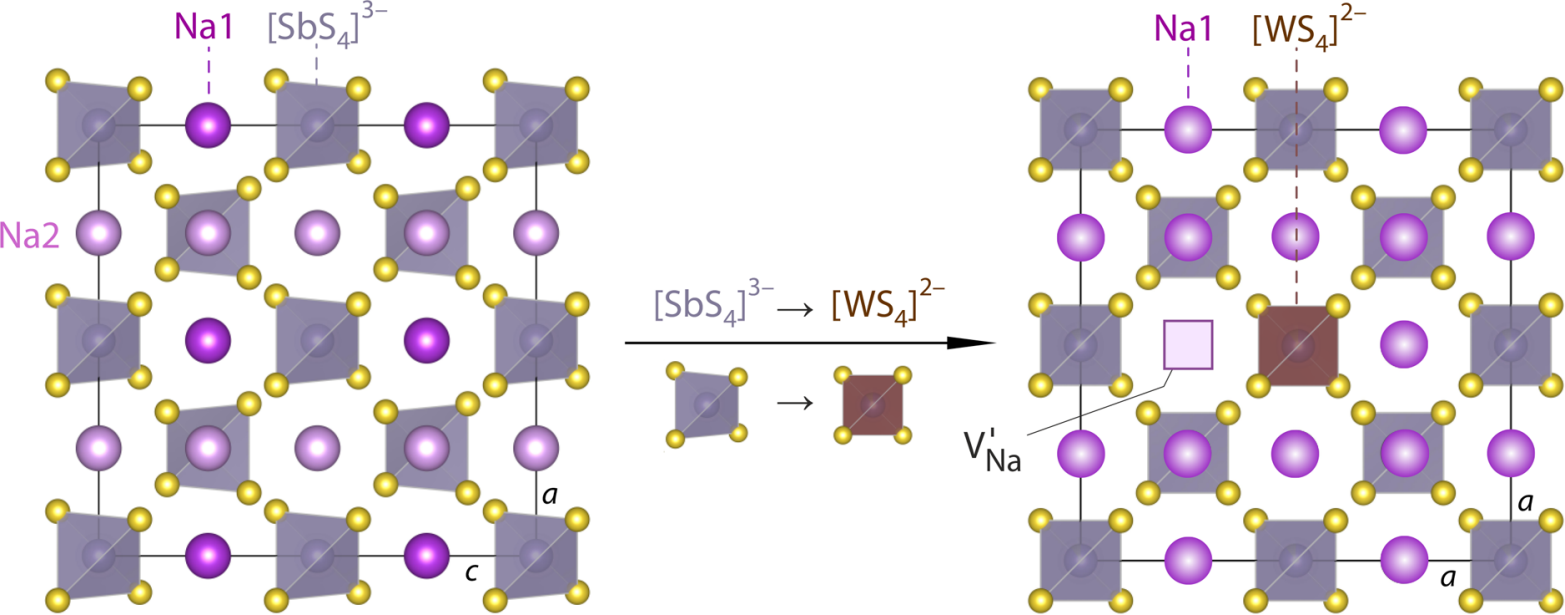
Crystal structure of Na_3_SbS_4_ crystallizing with tetragonal symmetry (view along the *b* axis). Replacing Sb^5+^ by W^6+^ introduces Na^+^ vacancies (V′_Na_) unlocking very high Na^+^ transfer through the material. This replacement is considered to change the overall (average) symmetry of the material from tetragonal to cubic, as indicated. In the present case, the symmetry shows a non-negligible degree of tetragonality even in our W-doped Na_3_SbS_4_.

## Results and Discussion

In this study, we investigated a W-doped Na_3_SbS_4_ sample with the composition Na_2.9_Sb_0.9_W_0.1_S_4_ prepared via conventional solid-state synthesis (see Supporting Information). X-ray diffraction (Figure S1), ^23^Na and ^121^Sb magic angle spinning NMR were employed to characterize both the overall and local structure (Figure S2) showing a non-negligible degree of tetragonality.

The corresponding broadband conductivity isotherms of W-doped Na_3_SbS_4_, constructed by plotting the real part of the complex conductivity vs frequency, σ′(ν), are shown in Figure 2a. As is best seen at low temperatures, the data are composed of three distinct regions, which we attribute to (A) electrode polarization effects, (B) regimes influenced by grain boundary effects, and (C) the electrical response representing pure intragrain ionic displacements (i.e., bulk ionic conduction, σ_bulk_ ∼ ω_e_). These assignments are based on capacitance values *C*_p_, which serve as reliable indicators to distinguish between bulk and total conductivities.^39,40^ Electrode polarization, with capacitance values on the order of μF, arises from the accumulation of charge carriers at the ion-blocking Au electrode applied to the pressed sample pellet. Ion-blocking at grain boundary regions is characterized by capacitance values around *C*_p_ (100 Hz, 113 K) ≈ 1 nF (see also Figure 3a), while the bulk response is clearly distinguished by capacitance values in the low pF range (≤10 pF). These values translate into the permittivity values shown in Figure 3a, which shows the real part *ε*′ of the complex permittivity as a function of frequency ν.^22^ The influence of bulk electrical processes on the permittivity becomes also apparent in the (dielectric) complex plane plot of Figure 3b, showing *ε*′ against the imaginary part of the permittivity *ε*″. In this representation, the bulk process causes a well-resolved semicircle that overlaps for the different isotherms. With increasing temperature, the process becomes masked by the overall conductivity.

**Figure 2:**
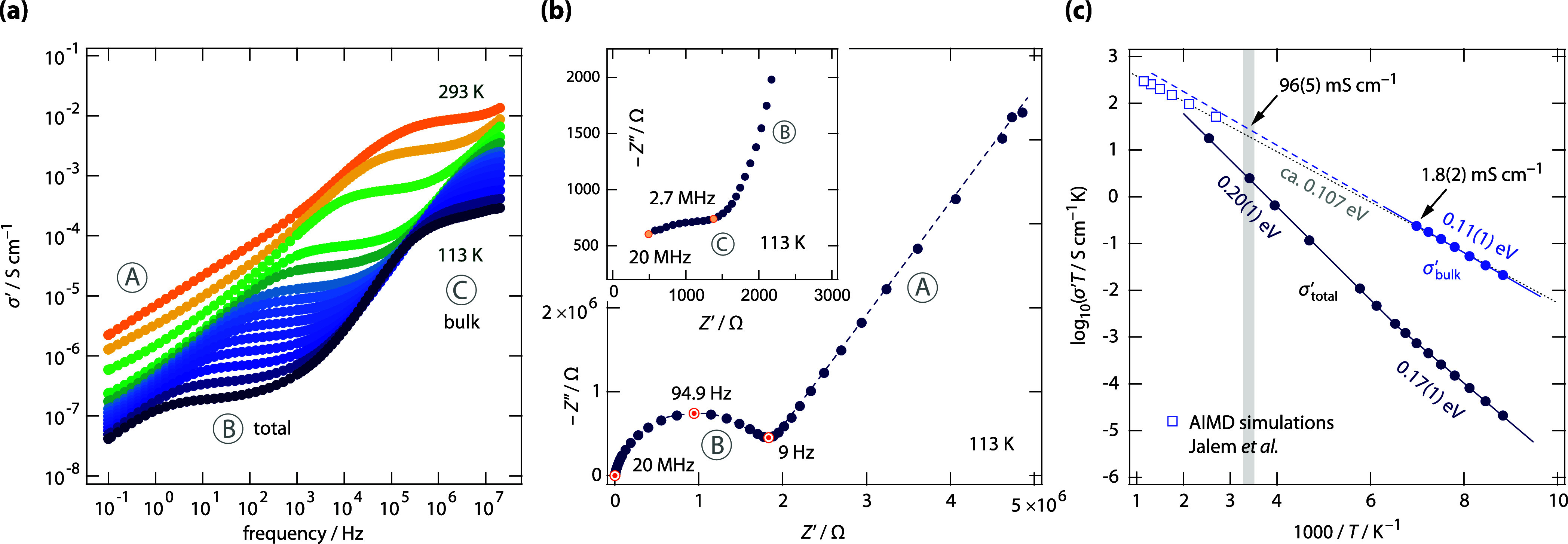
(a) Conductivity isotherms of polycrystalline Na_2.9_Sb_0.9_W_0.1_S_4_ recorded at temperatures ranging from 113 to 273 K. The three regions refer to electrode polarization (A), total conductivity (B) and bulk ionic transport (C). (b) Nyquist representation also revealing the distinct conductivity regions, the inset shows the bulk response. (c) Arrhenius plot showing the temperature dependence of the total (σ_total_) and bulk ionic conductivities (σ_bulk_). The lines show linear fits yielding the activation energies *E*_a, bulk_ (0.11 eV) and *E*_a, total_ (0.17 eV, 0.20 eV) as indicated. *E*_a, bulk_ = 110(10) meV excellently agrees with the value suggested by Jalem et al. from AIMD simulations (104(21) meV). Extrapolation of the bulk values points to conductivities reaching 96(5) mS cm^–1^ at ambient conditions (see the dashed line). The dotted line shows an Arrhenius line fit considering both the calculated and measured conductivities; the resulting activation energy is approximately 0.107 eV.

**Figure 3:**
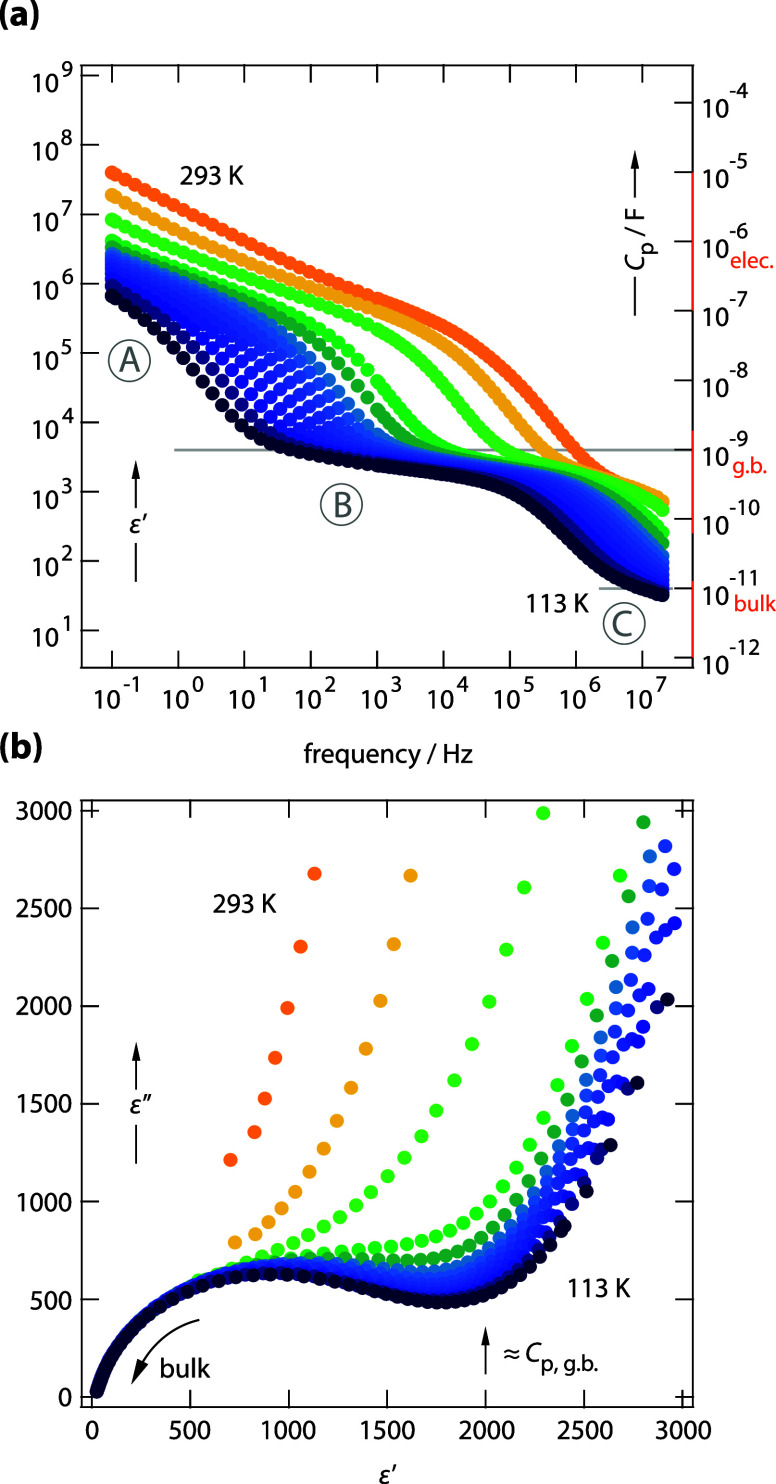
(a) Permittivity isotherms *ε*′(ν) of Na_2.9_Sb_0.9_W_0.1_S_4_ supporting the assignment of the three conductivity regions to electrode polarization, the electrical response including grain boundary contributions, and the pure (ionic) electrical permittivity due to intragrain (bulk) processes. For comparison, the right axis shows the capacitance *C*_p_ with the values for the three regions highlighted. (b) Permittivity complex plane plot, *ε*′(*ε*″), highlighting the bulk response at lower temperatures, high frequencies and *ε*′ < 100.

At sufficiently low temperatures, the three regions in conductivity spectroscopy can also be identified in the impedance Nyquist plot shown in Figure 2b, which depicts the imaginary part –*Z*″ of the complex impedance versus its real part *Z*′. The main depressed semicircle, whose intercept with the *Z*′ axis represents the total impedance, passes into a spike-like feature indicative of electrode polarization. This feature is typical for ionic conductors, where the ionic conductivity significantly exceeds any electronic contribution to the total conductivity σ_total_. Indeed, chronoamperometric measurements reveal that the electronic conductivity σ_eon_ is several orders of magnitude lower than σ_total_ (see Figure S3). In contrast to σ′(ν), the semicircle mirroring bulk ion transport is more difficult to see in the –*Z*″(*Z*′) complex plane representation. However, the inset in Figure 2b partly reveals this contribution, which corresponds to the high-frequency plateau (C) in Figure 2a.

In Figure 2c, we show the temperature dependence of both the total and bulk conductivities in an Arrhenius plot, where log_10_(σ_total_ *T*) and log_10_(σ_bulk_ *T*) are plotted against the reciprocal temperature (1/*T*). σ_total_ and σ_bulk_ have been read off from the plateaus (B) and (C) in Figure 2a, the approach is shown elsewhere.^41,42^ It uses the positions of the peaks of the loss angle on the frequency scale to determine ν belonging to σ_total_ and σ_bulk_, respectively. For the total conductivity, we observe a slight increase in activation energy from 0.17 to 0.20 eV around 160 K. The value of 0.17 eV aligns well with activation energies reported in the literature for ionic transport influenced by grain boundary effects (Figure 4).^16,17^ We believe that this feature depends on the specific synthesis conditions and is confined to grain-boundary properties. As shown below, the bulk ion dynamics exhibit a single Arrhenius behavior over a wide dynamic range spanning 4 decades. Considering that the sample for impedance measurements are prepared by cold pressing and subsequent sintering at 548 K (12 h, see Supporting Information) rather than by hot pressing, its total conductivity of 10 mS cm^–1^ at room temperature is approximately 3–4 times lower than the values reported by others for hot-pressed impedance samples (see Figure S4).^16,17,36^

**Figure 4:**
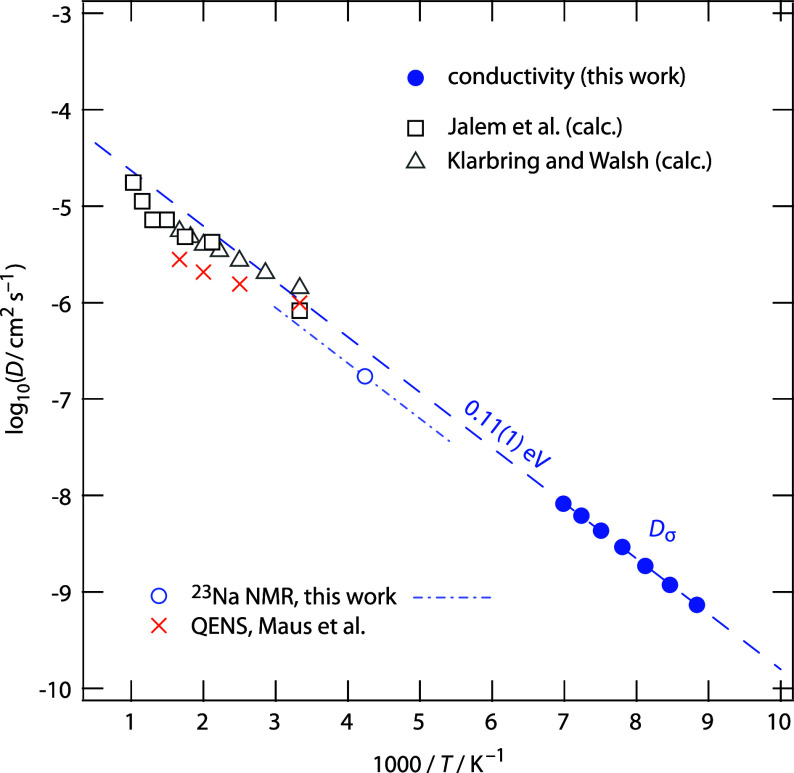
Comparison of Na^+^ ion diffusion coefficients of Na_2.9_Sb_0.9_W_0.1_S_4_, obtained by converting σ_bulk_ into *D*_σ_, with calculated self-diffusion coefficients (*D*_sd, calc_) from Klarbing and Walsh^37^ as well as diffusion coefficients from Jalem et al.^35^ (*D*_MD_). The dashed line shows the Arrhenius behavior of *D*_σ_, which excellently agrees with the calculated results when extrapolated to higher temperatures. For comparison, we also included self-diffusion coefficients from QENS as reported by Maus et al.^36^ The NMR diffusion coefficient has been extracted from preliminary ^23^Na NMR relaxation measurements ranging from 330 K down to 180 K (dashed-dotted line, Figure S5); see text for further details.

Bulk ionic transport in Na_2.9_Sb_0.9_W_0.1_S_4_ is nearly 3 orders of magnitude higher at lower temperatures. Even at a temperature as low as 143 K (−130 °C), the measured bulk conductivity remained remarkably high at 1.8 mS cm^– 1^ (see Figure 2c). The activation energy of 0.11(1) eV excellently agrees with values obtained from *ab initio* molecular dynamics (AIMD) simulations by Jalem et al. (0.104(21) eV).^35^ A very similar value (0.09 eV) has also been suggested by ^23^Na NMR relaxation measurements by Maus et al.^36^ Our own preliminary ^23^Na NMR spin–lattice relaxation measurements, which are exclusively sensitive to bulk ion dynamics, confirm this finding. These consistent results further support that the conductivities σ′(ν) of high-frequencies regime (C) in Figure 2a solely reflect intragrain Na^+^ ion dynamics.

When extrapolating the Arrhenius line describing bulk Na^+^ ion transport from low to higher temperatures, we find that σ_bulk_ reaches 96(5) mS cm^–1^ at 293 K. This value significantly exceeds the total conductivities of previously studied (hot-pressed) samples and positions Na_2.9_Sb_0.9_W_0.1_S_4_ among the fastest Na-ion conductors.^16,17^ Such high conductivities are otherwise observed only in certain high-temperature modifications of Ag^+^-conducting materials (e.g., AgI, RbAg_4_I_5_, Ag_3_SI), where extreme Ag^+^ mobility is attributed to a molten cation substructure, leading to *superionic* conductivity behavior. While α*-Ag_3_SI shows a conductivity of 38 mS cm^–1^ at room temperature,^43^ in α-RbAg_4_I_5_, conductivities as high as 250 mS cm^–1^ have been reported at 300 K,^24,44^ depending on the exact sample preparation conditions. A similar dependence on preparation and measuring conditions is observed for the conductivity of polycrystalline α-AgI, which, however, shows conductivities higher than 1 S cm^–1^ only above the α-β phase transition at 146 °C.^45,46^ At room temperature, the ionic conductivity of β-AgI is clearly lower than 0.1 mS cm^–1^.^45^ As has been shown by Tatsumisago et al.,^45^ only if stabilized as α-AgI nanoparticles in a glassy matrix, conductivities of 30 mS cm^–1^ can be reached at ambient conditions. This value and the conductivity of Na-β″-alumina (10 mS cm^–1^)^24^ are, however, still significantly lower than that for Na_2.9_Sb_0.9_W_0.1_S_4_.

Assuming that all Na^+^ ions within the unit cell of Na_2.9_Sb_0.9_W_0.1_S_4_ take part in extremely fast ion dynamics, we can estimate the number density of charge carriers per volume *N*_c_ to convert σ_bulk_(293 K) into a solid-state (or charge) diffusion coefficient *D*_σ_, which is given by *D*_σ_ = σ_bulk_ *k*_B_ *T*/(*N*_c_ *q* ^2^) with the Boltzmann constant *k*_B_ and *q* denoting the charge of the Na^+^ ions. With *N*_c_ = 1.55 × 10^28^ m^–3^, we obtain an extremely high diffusion coefficient of *D*_σ_ = 9.8(2) × 10^–11^ m^2^ s^–1^ (see Figure 4). This value perfectly agrees with that reported by Jalem et al.^35^ obtained from AIMD simulations (*D*_MD_(300 K) = 8.2 × 10^–11^ m^2^ s^–1^) for Na_2.875_Sb_0.875_W_0.125_S_4_. For this composition, the authors report a conductivity of approximately 80.6 mS cm^–1^ at ambient temperature,^35^ being close to the value proposed in our study.

In Figure 4, the diffusion coefficients *D*_σ_ of this work are compared with calculated diffusion coefficients from Jalem et al. (*D*_MD_)^35^ and from Klarbring and Walsh (*D*_sd, calc_).^37^ The dashed line shows the Arrhenius line of the solid-state diffusion coefficients extracted from σ_bulk_; its extrapolation toward higher temperatures shows excellent agreement with the calculated values. In general, the microscopic self-diffusion coefficient *D*_sd_ is related to *D*_σ_ via *D*_sd_ = (*H*_r_/*f*) × *D*_σ_ with the Haven ratio *H*_r_ connecting the macroscopic tracer diffusion coefficient *D*_t_ with *D*_σ_ (*D*_t_ = *H*_r_ *D*_σ_) and the correlation factor *f* given by *f* = *D*_t_/*D*_sd_. Depending on the particular interpretation, *H*_r_ might already account for *f*. For comparison, in Figure 4, we included a preliminary self-diffusion coefficient *D*_NMR_ determined by ^23^Na nuclear magnetic resonance (NMR) measurements (Figure S5). The ratio *D*_NMR_/*D*_σ_ yields *H*_r_/*f* = 0.58 at 235 K. This ratio decreases to 0.36 at higher temperatures (*T* ≥ 500 K) if we compare diffusion coefficients *D*_QENS_ from quasielastic neutron scattering (QENS)^36^ with the extrapolated *D*_σ_ values from this work. So far, experimental tracer diffusion coefficients have not been reported for polycrystalline Na_2.9_Sb_0.9_W_0.1_S_4_. Nevertheless, Figure 4 once again underscores the excellent agreement between measured and calculated dynamic parameters governing Na^+^ ion transport and diffusivity in Na_2.9_Sb_0.9_W_0.1_S_4_. The activation energy determined by variable-temperature ^23^Na NMR spin–lattice relaxation measurements (0.105(5) eV; see Figure S5 and details in the Supporting Information), which reflects diffusion-induced ^23^Na NMR relaxation from 180 to 330 K (dashed-dotted line in Figure 4), is in excellent agreement with the activation energy obtained from conductivity spectroscopy (measured up to 143 K). With a Na^+^ diffusion coefficient of 10^–10^ m^2^ s^–1^, Na_2.9_Sb_0.9_W_0.1_S_4_ exhibits an unprecedentedly fast Na^+^ transport process, establishing it as one of the best nonmetallic Na-ion conductors reported to date.

## Conclusions

We investigated the ionic transport properties of W-doped Na_3_SbS_4_ (Na_2.9_Sb_0.9_W_0.1_S_4_), a promising solid electrolyte with an *electronic* conductivity σ_eon_ of 10^–8^ S cm^–1^ (293 K) for all-solid-state sodium-ion batteries. Using broadband impedance spectroscopy at low temperatures, we isolated the intrinsic Na^+^ bulk *ionic* conductivity, confirming that previously reported values were influenced by grain boundary resistances. Notably, even at a temperature as low as 143 K (−130 °C), the experimental bulk conductivity remains high at 1.8 mS cm^– 1^, underscoring the outstanding ionic transport properties of Na_2.9_Sb_0.9_W_0.1_S_4_. Extrapolating our findings to room temperature (293 K), we determined an exceptionally high bulk ionic conductivity σ_bulk_ reaching 96 mS cm^–1^ and a Na^+^ diffusion coefficient of 10^–10^ m^2^ s^–1^. These values, along with the activation energy (0.11 eV) of the bulk ionic conductivity, show excellent agreement with previously reported calculation results. Our current findings indeed establish vacancy-rich Na_2.9_Sb_0.9_W_0.1_S_4_, as one of the fastest known nonmetallic Na-ion conductors, comparable to or exceeding the best Li^+^ ion-conducting thiophosphates, highlighting its potential for next-generation sodium-based energy storage technologies. The finding that bulk ion dynamics are much faster than previously anticipated opens up the possibility of using grain-boundary engineering to enhance total conductivity to levels approaching those of the bulk. Soft sintering steps, the addition of sintering aids, and refined synthesis protocols may help achieve this.

## Experimental Section

Polycrystalline Na_2.9_Sb_0.9_W_0.1_S_4_ was synthesized via conventional solid-state methods. A mixture of Na_2_S, Sb_2_S_3_, WS_2_, and S (molar ratio 1.45:0.45:0.1:1) was hand-ground and milled for 5 h at 600 rpm in a ZrO_2_ beaker (ball-to-powder ratio: 11:1). The powder was pelletized and sealed in evacuated quartz tubes, followed by heat treatment at 275 °C for 12 h (5 °C min^–1^), see Supporting Information for further details and sample characterization by X-ray powder diffraction and high-resolution ^23^Na and ^121^Sb NMR. Ionic conductivities were determined by impedance and conductivity spectroscopy using a Novocontrol Concept 80 broadband spectrometer, see Supporting Information. Electronic conductivities were measured via potentiostatic chronoamperometry, and Na^+^ self-diffusivity was probed by variable-temperature ^23^Na NMR spin–lattice relaxation measurements; see also Supporting Information for further details.

## Supplementary Material



## Notes

Experimental details on sample preparation, X-ray diffraction data, conductivity and polarization measurements, high-resolution NMR spectra, NMR relaxation data (PDF)
