# Supplementary material for: Unveiling Ultra-High Ionic Conductivity in W‑Doped Na3SbS4: Grain Boundary Effects and Pure Bulk Transport
Source: J Am Chem Soc. 2025 May 29;147(23):20092–7. doi: 10.1021/jacs.5c05842 (PMC12164346; doi:10.1021/jacs.5c05842)
Supplement: Supplementary file 1 [file ja5c05842_si_001.pdf]

Supporting Information

Unveiling Ultra-High Ionic Conductivity in W-Doped Na_3SbS_4: Grain Boundary Effects and Pure Bulk Transport

Jana Königsreiter, Bernhard Gadermaier, and H. Martin R. Wilkening*

Institute for Chemistry and Technology of Materials (NAWI Graz), Graz University of Technology, AT-8010 Graz, Austria

E-mail: wilkening@tugraz.at

Experimental

Sample Preparation of Polycrystalline $Na_{2.9}Sb_{0.9}W_{0.1}S_4$

The synthesis of $Na_{2.9}Sb_{0.9}W_{0.1}S_4$ in this work was inspired by Hayashi and co-workers.[1] To synthesize polycrystalline $Na_{2.9}Sb_{0.9}W_{0.1}S_4$, the starting materials Na_2S (Sigma-Aldrich, no specified purity), Sb_2S_3 (Sigma-Aldrich, 99.995%), WS_2 (Sigma-Aldrich, 99%), and S (Sigma-Aldrich, 99.998%) were weighed in a molar ratio of 1.45:0.45:0.1:1. The educts were briefly hand-ground before being transferred into a 45 mL ZrO_2 milling beaker together with 180 ZrO_2 balls (ball-to-powder ratio: 11:1) and subsequently milled for 5 h at 600 rpm. The resulting powder was pressed into pellets (5 mm in diameter, 1 ton) and fire-sealed in evacuated quartz tubes. A subsequent heat treatment was performed at 275 °C for 12 h with a heating rate of 5 °C min^{-1}. All synthesis steps, except for the heat treatment, were carried out under an inert Ar atmosphere (GS Glovebox, $O_2 < 1$ ppm, $H_2O < 1$ ppm).

X-Ray Powder Diffraction

X-ray powder diffraction measurements were carried out using a Rigaku MiniFlex diffractometer with Cu-Kα radiation ($\lambda = 1.54059$ Å) and a D/teX Ultra silicon strip detector. To prevent contamination by air or moisture, an air-tight sample holder was used. The measurements covered a 2θ range from 10° to 70°, with a scanning speed of 2° min^{-1} and a step size of 0.01°. For Rietveld crystal structure refinements (see Figure S1), the Malvern Panalytical X'Pert HighScore Plus software was used, along with reference diffraction patterns obtained from the Inorganic Crystal Structure Database (ICSD).

Supporting Information

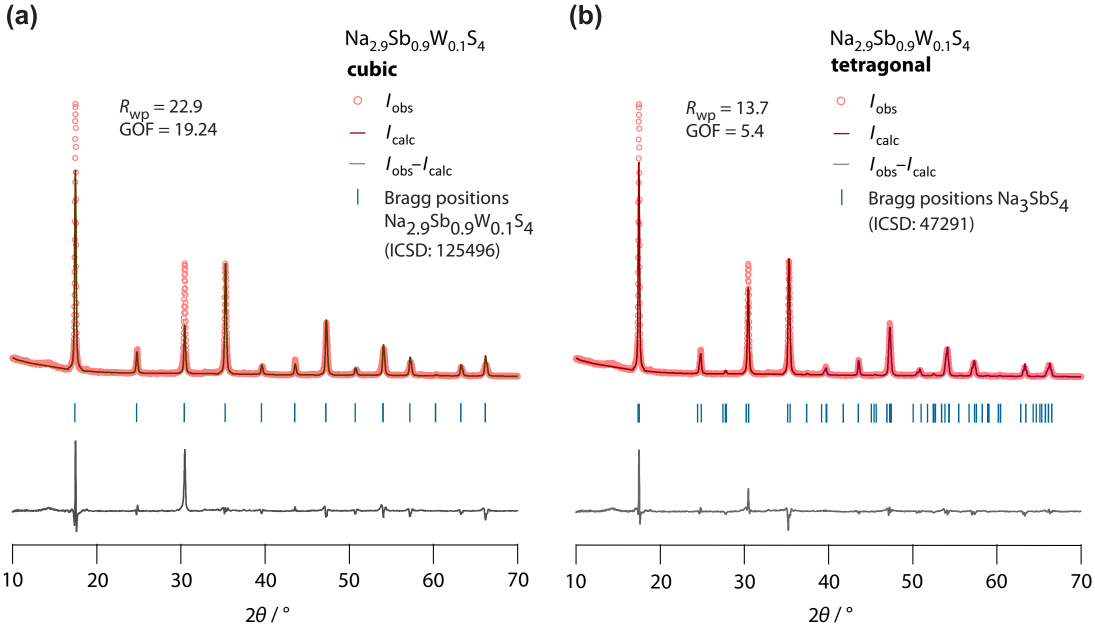

Figure S1: Rietveld refinement of the powder diffraction pattern of $Na_{2.9}Sb_{0.9}W_{0.1}S_4$. Refinement allowing a certain degree of tetragonality (b) resulted in a better overall result, as compared to an analysis restricted to cubic symmetry (a).

High-Resolution NMR

Magic-angle spinning (MAS) NMR spectra of ^{23}Na (132.3 MHz) and ^{121}Sb (119.7 MHz) were recorded using a Bruker Avance III spectrometer connected to a shimmed magnet with a nominal field of 11.7 T. The powdered sample was pressed into a 2.5-mm zirconia rotor equipped with a Vespel cap and measured at a rotation speed of 25 kHz at ambient temperature. A single 90° (π/2) pulse with a sufficiently long delay time was used, and NMR spectra were obtained by Fourier transformation of the recorded free induction decays without further manipulation of the time-domain signal. For the ^{23}Na spectra, solid (polycrystalline) NaCl (0 ppm) was used as a reference. ^{121}Sb measurements were referenced to an arbitrary spectrum reference frequency of $-12{,}684.3$ Hz.

Conductivity Spectroscopy

For impedance spectroscopy, the high-density pellets resulting from heat treatment were equipped with 50 nm ion-blocking Au electrodes on both sides. The electrodes were sputtered using a Leica EM SCD050, placed inside a glovebox. The complex impedance data were recorded in the frequency range of 100 mHz to 20 MHz using a Novocontrol Concept 80 broadband impedance spectrometer (WinDeta software) under an inert gas stream (N_2). The temperature ranged from 120 °C to –160 °C with a varying step size between different temperatures. Temperature control was performed using a Novocontrol QUATRO cryo-system.

Supporting Information

Potentiostatic Polarization Measurements

The electronic conductivity of pellets from the same batch for impedance spectroscopy was determined using a Versa STAT3 potentiostat. A constant potential of 0.1 V was applied, and the current response was recorded over a time period of 10 h for each temperature (20 °C, 60 °C, and 100 °C).

Structural Analysis by XRD and NMR

To determine the average structure of the synthesized $Na_{2.9}Sb_{0.9}W_{0.1}S_4$, X-ray powder diffraction followed by subsequent Rietveld refinement was used. In Figure S1, the refinements are shown with either a cubic or a tetragonal Na_3SbS_4/$Na_{2.9}Sb_{0.9}W_{0.1}S_4$ reference. As can be seen, no significant peak splitting at the major reflections is observed, suggesting, at first glance, that the prepared material exhibits a cubic structure. The transition from the tetragonal $Na_3SbS_4$to cubic W-doped Na_3SbS_4has been reported by others, where W-doped samples with a tungsten content >10% show an average cubic structure.[1,2] Here, Rietveld refinement resulted in a better fit with the tetragonal structure. Using the determined lattice parameters c and a; here, a ($c : a$) ratio of 1.006 was calculated, making the synthesized sample 'pseudo-cubic' or almost cubic.

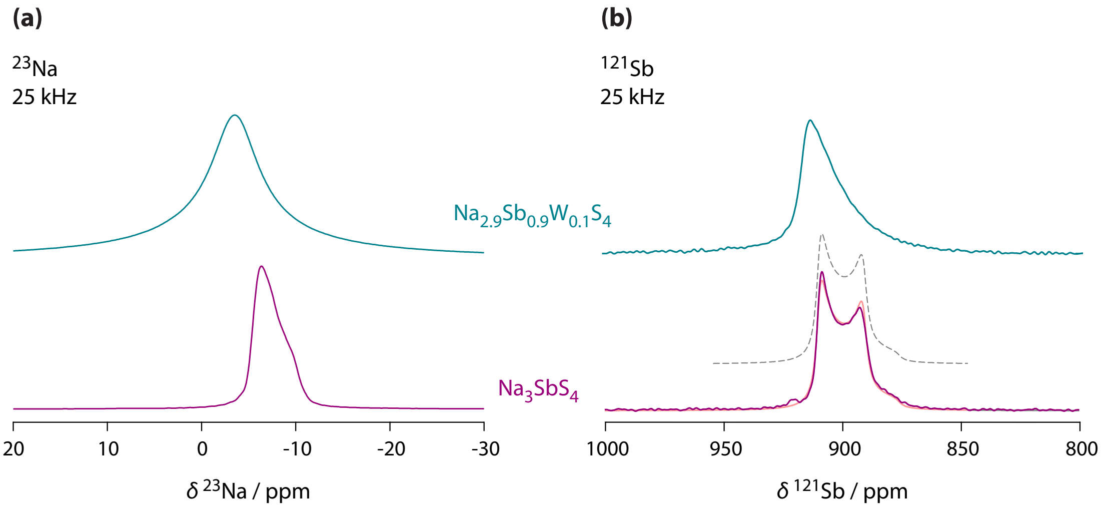

Figure S2: ^{23}Na and ^{121}Sb MAS NMR spectra of $Na_{2.9}Sb_{0.9}W_{0.1}S_4$. Spectra for W-free Na_3SbS_4 are shown for comparison. The dashed line shows a simulation of the ^{121}Sb MAS NMR with a single set of quadrupolar parameters representing the Sb position in the sulfide.

As magic-angle spinning (MAS) NMR is sensitive to magnetically distinct sites in the material, ^{23}Na and ^{121}Sb MAS NMR were used to investigate the local environments of the two kinds of nuclei. In Figure S2, the ^{23}Na and ^{121}Sb MAS NMR spectra of $Na_{2.9}Sb_{0.9}W_{0.1}S_4$ are compared to those of pristine W-free Na_3SbS_4 synthesized in the same way as a reference. The ^{23}Na NMR spectra show only one signal, which is expected for both tetragonal and cubic structures, as fast ionic exchange processes between different Na sites would cause averaging of the corresponding signals. For the W-doped sample, a slightly broader and more anisotropic NMR line with a significant change in the chemical shift to −3.5 ppm is observed. Most likely, the introduction of

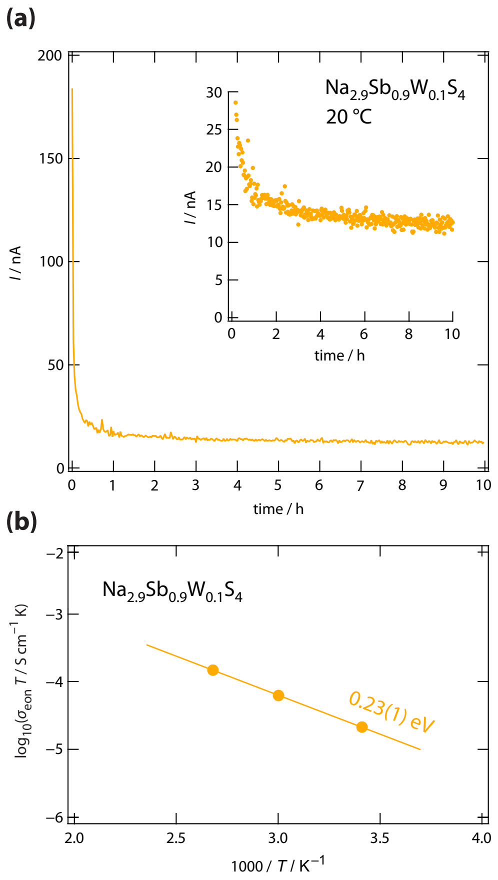

Figure S3: Polarisation curve (a) and Arrhenius plot (b) of electronic conductivities of $Na_{2.9}Sb_{0.9}W_{0.1}S_4$. The residual current of 12.5 nA translates into $\sigma_{eon} = 7.26 \times 10^{-8}$ S cm^{-1} at ambient temperature.

tungsten leads to an overall change in the local (and averaged) magnetic environment of $^{23}Na^+$ in cation-mixed $Na_{2.9}Sb_{0.9}W_{0.1}S_4$. Similarly, the ^{121}Sb NMR spectra point to only one Sb position. While the corresponding spectrum of Na_3SbS_4 represents an ideal NMR line of the quadrupolar nucleus ^{121}Sb (spin-quantum number $I = 5/2$) under MAS conditions, an anisotropic NMR line is observed in the tungsten-doped material, again pointing to the successful introduction of W and local structural distortions of the framework structure. The dashed line in Figure S2(b) shows a proper simulation of the spectrum with a single set of quadrupolar parameters. The quadrupole coupling constant turned out to be 919.1 kHz. For the asymmetry parameter we found $\eta_q = 0$.

Polarisation Measurements

To assess whether the high total conductivity determined by impedance spectroscopy is influenced by electronic contributions, potentiostatic polarization measurements were performed. The electronic conductivity, σ_{eon}, can be considered low, as it is 5–6 orders of magnitude lower than the ionic conductivity.[3] The polarization curve of $Na_{2.9}Sb_{0.9}W_{0.1}S_4$ at 20 °C, shown in Figure S3(a),

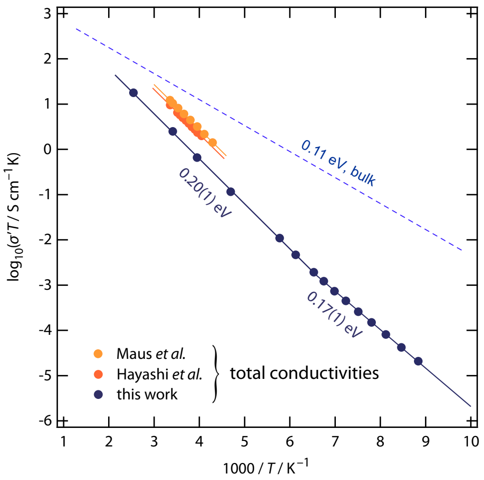

Figure S4: A comparison of bulk and total conductivities of $Na_{2.9}Sb_{0.9}W_{0.1}S_4$ from both this study and literature as indicated.[1,2] The dashed lines refer to the bulk conductivities (and diffusion coefficients) probed in this study, see Figure 2c (and Figure 4).

yields an electronic conductivity of 7.26×10^{-8} S cm^{-1}. This value is significantly lower than the measured total conductivity, confirming that Na^{+} ions serve as the primary charge carriers. Similar electronic contributions to the overall conductivity in W-doped Na_3SbS_4 at room temperature have been reported by others.[1,4]

Analogous to impedance spectroscopy, the activation energy of the electronic conductivity, σ_{eon}, was determined using an Arrhenius analysis of temperature-dependent measurements. As shown in Figure S3(b), the activation energy was found to be 0.23(1) eV.

Total Conductivities

Total conductivities of $Na_{2.9}Sb_{0.9}W_{0.1}S_4$ as a function of temperature are shown in Figure S4. Literature data are included to illustrate the influence of sample preparation conditions on grain-boundary resistance.

Spin-Lattice Relaxation NMR

^{23}Na NMR spin-lattice relaxation rates were measured as a function of temperature using the saturation recovery pulse sequence.[5] Details on this type of measurement can be found elsewhere.[6] The rates were recorded on a Bruker Avance III spectrometer connected to a shimmed cryomagnet with a nominal field strength of approximately 7.04 T, corresponding to a Larmor (resonance) frequency $\omega_0/2\pi$ of 79.35 MHz. A Bruker broadband probe (80 mm in diameter) with a Teflon sample chamber was used to acquire the signals. The sample temperature was controlled via a stream of heated dinitrogen gas and regulated using a Eurotherm temperature controller, with an accuracy of ± 2 K.

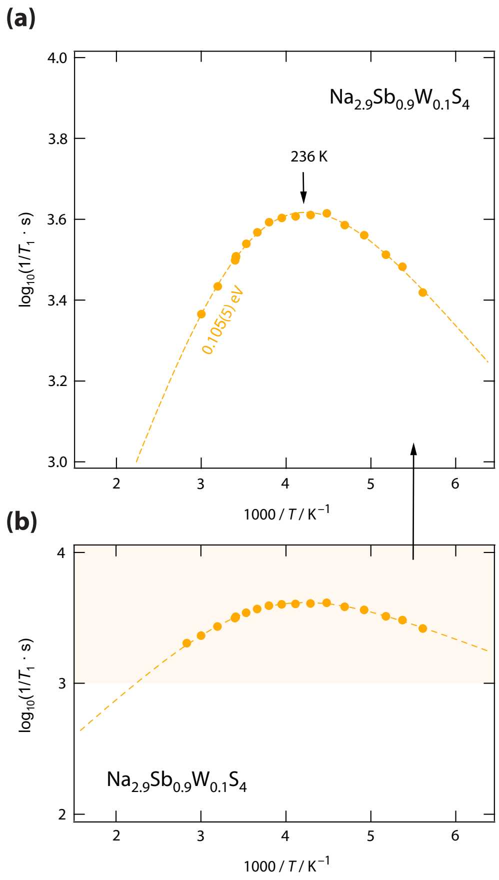

Figure S5: (a) Magnified view of the diffusion-induced rate peak of $Na_{2.9}Sb_{0.9}W_{0.1}S_4$ seen in variable-temperature ^{23}Na NMR spin-lattice relaxation measurements. The rate T_1^{-1} is plotted as a function of inverse temperature. The activation energy for the high-temperature flank of the peak was derived from the BPP model (ca. 0.11 eV) and excellently agrees with that probed by conductivity spectroscopy at lower temperatures. (b) The ^{23}Na relaxation rate maximum of $Na_{2.9}Sb_{0.9}W_{0.1}S_4$ shown with a differently scaled axis to highlight the broad overall shape of the peak.

In Figure S5, the diffusion-induced NMR relaxation rates are analyzed using an Arrhenius plot. At the temperature corresponding to the relaxation rate maximum, the mean Na^+ residence time is given by the condition $\tau = 0.62/\omega_0$. The associated jump rate can be converted into a diffusion coefficient via $D_{\mathrm{NMR}} = a^2/(2d\tau)$, where a is the jump distance determined from the crystal structure.[4] For three-dimensional diffusion ($d = 3$), this yields the coefficient shown in Figure 4 (see main text).

The solid line in Figure S5 represents the result of a BPP analysis, which approximates the shape of the asymmetric peak using a Lorentzian-type spectral density function according to Bloembergen, Purcell, and Pound.[7] Across the investigated temperature range, the model provides a satisfactory fit and yields an activation energy of 0.105(5) eV, which is in excellent agreement with the value determined by conductivity spectroscopy (see Figure 4).

Supporting Information

References

(1) Hayashi, A.; Masuzawa, N.; Yubuchi, S.; Tsuji, F.; Hotehama, C.; Sakuda, A.; Tatsumisago, M. A Sodium-Ion Sulfide Solid Electrolyte with Unprecedented Conductivity at Room Temperature. *Nature Commun.* **2019**, *10*, 5266.

(2) Maus, O.; Agne, M. T.; Fuchs, T.; Till, P. S.; Wankmiller, B.; Gerdes, J. M.; Sharma, R.; Heere, M.; Jalarvo, N.; Yaffe, O.; Hansen, M. R.; Zeier, W. G. On the Discrepancy between Local and Average Structure in the Fast Na^+ Ionic Conductor $Na_{2.9}Sb_{0.9}W_{0.1}S_4$. *J. Am. Chem. Soc.* **2023**, *145*, 7147–7158.

(3) Philipp, M.; Gadermaier, B.; Posch, P.; Hanzu, I.; Ganschow, S.; Meven, M.; Rettenwander, D.; Redhammer, G. J.; Wilkening, H. M. R. The Electronic Conductivity of Single Crystalline Ga-Stabilized Cubic $Li_7La_3Zr_2O_{12}$: A Technologically Relevant Parameter for All-Solid-State Batteries. *Adv. Mater. Interf.* **2020**, *7*, 2000450.

(4) Fuchs, T.; Culver, S. P.; Till, P.; Zeier, W. G. Defect-Mediated Conductivity Enhancements in $Na_{3-x}Pn_{1-x}W_xS_4$ (Pn = P, Sb) Using Aliovalent Substitutions. *ACS Energy Lett.* **2020**, *5*, 146–151.

(5) Epp, V.; Gün, O.; Deiseroth, H.-J.; Wilkening, M. Long-Range Li^+ Dynamics in the Lithium Argyrodite Li_7PSe_6 as Probed by Rotating-Frame Spin-Lattice Relaxation NMR. *Phys. Chem. Chem. Phys.* **2013**, *15*, 7123–7132.

(6) Stainer, F.; Gadermaier, B.; Kügerl, A.; Ladenstein, L.; Hogrefe, K.; Wilkening, H. M. R. Fast Na^+ ion dynamics in the Nb^{5+} bearing NaSICON $Na_{3+x-z}Nb_zZr_{2-z}Si2+xP_{1-x}O_{12}$ as probed by ^{23}Na NMR and conductivity spectroscopy. *Solid State Ionics* **2023**, *395*, 116209.

(7) Bloembergen, N.; Purcell, E. M.; Pound, R. V. Relaxation Effects in Nuclear Magnetic Resonance Absorption. *Phys. Rev.* **1948**, *73*, 679–712.
